# Analysis of primary risk factors for oral cancer from select US states with increasing rates

**DOI:** 10.1186/1617-9625-8-5

**Published:** 2010-02-23

**Authors:** Anthony Bunnell, Nathan Pettit, Nicole Reddout, Kanika Sharma, Susan O'Malley, Michelle Chino, Karl Kingsley

**Affiliations:** 1 Department of Biomedical Sciences, School of Dental Medicine, University of Nevada, Las Vegas, USA; 2 College of Arts and Sciences, University of Pennsylvania, Philadelphia, USA; 3 Department of Environmental and Occupational Health, School of Community Health Sciences, University of Nevada, Las Vegas, USA

## Abstract

**Objectives:**

To examine the primary risk factor for oral cancer in the US, smoking and tobacco use, among the specific US states that experienced short-term increases in oral cancer incidence and mortality.

**Methods:**

Population-based data on oral cancer morbidity and mortality in the US were obtained from the National Cancer Institute's (NCI) Surveillance, Epidemiology, and End Results (SEER) database for analysis of recent trends. Data were also obtained from the Centers for Disease Control and Prevention (CDC) Behavioral Risk Factor Surveillance System (BRFSS) to measure current and former trends of tobacco usage. To comprehensive measures of previous state tobacco use and tobacco-related policies, the Initial Outcomes Index (IOI, 1992-1993) and the Strength of Tobacco Control index (SoTC, 1999-2000) were also used for evaluation and comparison.

**Results:**

Analysis of the NCI-SEER data confirmed a previous report of geographic increases in oral cancer and demonstrated these were state-specific, were not regional, and were unrelated to previously observed increases among females and minorities. Analysis of the CDC-BRFSS data revealed these states had relatively higher percentages of smokers currently, as well as historically. In addition, analysis of the IOI and SoTC indexes suggest that many factors, including cigarette pricing, taxes and home or workplace bans, may have had significant influence on smoking prevalence in these areas. Trend analysis of these data uncovered a recent and significant reversal in smoking rates that suggest oral cancer incidence and mortality may also begin to decline in the near future.

**Conclusion:**

Due to the rising costs of health care in the US and the limited resources available for health prevention efforts, it is essential to organize and direct more effective efforts by public health officials and epidemiologists, as well as funding from local, state and federal governments, to reduce and eliminate identified health disparities. This study provides evidence how these efforts may be directed to specific geographic areas, and towards the white males, previously thought to be unaffected by the increases in oral cancer among females and minorities.

## Background

Although oral cancer incidence and mortality rates have increased worldwide, these rates have been slowly and steadily declining among the US population over the past thirty years [1,2]. Despite the overall declining trends of oral cancer in the US, these declines are neither consistent nor uniform within this population [3,4]. Researchers have found that the incidence among specific demographic subgroups may have actually increased over this same time period [5-7]. Recent studies have shown that rates of oral cancer have been steadily declining among males, but have risen sharply among females [8]. More specifically, the declining rates observed among males were specific mainly to white males, while increasing incidence was found among minorities, and black males, in particular [8]. A new study of oral cancer epidemiology has found that increases in incidence and mortality may also exhibit geographic specificity within the US [9], providing compelling rationale to analyze the risk factors for oral cancer within these specific geographic areas and among these specific demographic subgroups.

Oral cancer incidence and mortality are correlated strongly with two major risk factors, tobacco use - consisting primarily of smoking in the US, and to a lesser extent, heavy alcohol use, which together account for the overwhelming majority of cases [10]. A recent study of smoking and tobacco use in the US found that rates declined sharply among males between 1965 and 1990, while the rates among females and minorities had less pronounced declines, and in some instances, may have increased [11]. In fact, more recent studies provide strong evidence that increasing usage of non-traditional forms of tobacco in the US, such as cigars and water pipe smoking, have become increasingly popular among females and minorities [12]. Although many studies have found correlations and linkages between increased workplace participation and social mobility, as well as acceptance and availability of tobacco products with the increasing rates of oral cancer among females and minorities, no studies to date have yet examined the relationship between increasing rates of oral cancer in a small subset of US states and the primary risk factors for oral cancer.

A review of oral cancer epidemiology in Europe revealed morbidity and mortality have been steadily decreasing since the early 1980s, similar to the trends observed in the US [13]. Temporal and geographic patterns, however, have demonstrated increasing oral cancer rates among specific eastern European countries following the disintegration and dissolution of the Soviet Union [14,15]. These studies have demonstrated the increases were highly correlated to changes in exposure to the primary risk factors for oral cancer, including tobacco and alcohol, which became more readily available during this time [14,15]. Although no analogous geopolitical events have precipitated rapid, sharp increases in the availability of either tobacco or alcohol within these select US states with increasing oral cancer rates, significant differences in cigarette pricing and taxes, as well as specific laws regarding smoking bans, may have created state-specific environments that influence the prevalence of these oral cancer risk factors over time.

This study sought to examine the primary risk factors for oral cancer, focusing specifically on tobacco use and smoking prevalence, among the US states recently found to have increasing short-term oral cancer incidence and mortality rates, including Nevada, North Carolina, Iowa, Ohio, Maine, Idaho, North Dakota and Wyoming [9]. More specifically, the working hypothesis for this study was that state-specific environmental factors may have led to increased tobacco use within these states. Data from the National Cancer Institute (NCI) Surveillance, Epidemiology and End Results (SEER) database, and the Behavioral Risk Factor Surveillance System (BRFSS), supported by the Centers for Disease Control and Prevention (CDC), were used to access and generate oral cancer statistics and comparisons of risk factor prevalence in these specific US states, over time. The identification of states, regions, or geographic areas with increased risk for oral cancer, as well as increased morbidity and mortality, is important because these represent sites where public health education and prevention efforts could be more effectively focused to improve health outcomes and reduce health disparities.

## Methods

### Mortality data: Surveillance, Epidemiology, and End Results (SEER)

Population-based data on oral cancer in the US were obtained from the Surveillance, Epidemiology, and End Results (SEER) program. SEER provides cancer incidence and survival data from population-based cancer registries, representing approximately 25% of the US population [16]. All oral cancer statistics in this report are based on SEER incidence and National Center for Health Statistics (NCHS) mortality statistics, which consisted of cancers of the oral cavity, pharynx and lip [17]. Oral cancer mortality rates between 1975 and 2005 were also obtained from SEER for each year, age-adjusted to the year 2000 standard US population. Deaths qualified for inclusion in SEER oral cavity and pharyngeal cancer if the underlying cause of death was specific for head and neck cancers [18]. The overall mortality trends over time were calculated and graphed based on available data from 1981-2005.

### Annual percent change (APC) for selected US states

Recent trend data in death rates from oral cancer in individual US states were calculated by the National Cancer Institute (NCI) SEER*Stat using data provided by the National Vital Statistics System public use data file (SEER) and from the State Cancer Registries using the Joinpoint Regression program and are expressed as the annual percent change (APC) over the reported trend period (1999-2003, for example) for selected US states. These states included Nevada (NV), North Carolina (NC), Iowa (IA), Ohio (OH), Maine (ME), Idaho (ID), North Dakota (ND), Wyoming (WY), Arizona (AZ), California (CA), Oregon (OR) and Utah (UT). Trends calculated using the Joinpoint Regression statistical software program model the natural logarithm of the rates, identifying years at which any given trend changes, connecting these years graphically by a series of straight line segments [19,20]. Current annual death rates of oral cancers from individual US states were similarly obtained and the most recent data available (2003, 2004 or 2005) at the time of article preparation were reported.

### Risk factor data: Behavioral Risk Factor Surveillance System (BRFSS)

Historical risk behavior data for tobacco use from selected US states were obtained from the Behavioral Risk Factor Surveillance System (BRFSS). BRFSS is among the largest health surveillance and survey systems, responsible for tracking data monthly and reporting health conditions and risk behaviors from all US states, the District of Columbia, Puerto Rico, the U.S. Virgin Islands, and Guam, since 1984 [21]. BRFSS is part of the National Center for Chronic Disease Prevention and Health Promotion, sponsored by the Centers for Disease Control and Prevention (CDC). Data included four level smoking status (Smoke Every Day; Smoke Some Days; Former Smoker; Never Smoked), and adults who are current smokers. Temporal data files were available for all states after 2001, and from selected states dating from 1984.

### Initial Outcomes Index (IOI) and Strength of Tobacco Control (SoTC)

State-specific data and rankings that form the US State Tobacco Control Initial Outcomes Index (IOI) were obtained from a previous report [22]. Measures used to generate the IOI index included smoking prevalence, computed as the percentage of current smokers who indicated at the time of the survey they smoked either every day or some days, per capita cigarette consumption, computed using total number of packs removed and sold in any given month divided by the US Bureau of Census estimates for state population aged 18 years or older at the time of the survey, weighted averages for cigarette prices during the time period analyzed, and the prevalence of workplace and home smoking bans. For the index factors (cigarette price per pack, workplace smoking bans among ever smokers and home smoking bans among ever smokers) *z *scores were calculated and summed to form a tobacco control IOI, which was correlated with adult smoking prevalence and the point estimate of per capita cigarette consumption. Similarly, state-specific data and rankings from 1999-2000 form the standardized Strength of Tobacco Control (SoTC) index, which were also obtained from previous reports [23,24]. Positive IOI or SoTC index scores indicate relatively robust state tobacco controls, including smoking bans, and generally reflect higher cigarette prices and taxes, while negative index scores indicate states with weaker tobacco controls, fewer smoking bans and comparatively lower cigarette prices and taxes.

## Results

### Surveillance Epidemiology and End Results (SEER)

**Figure 1:**
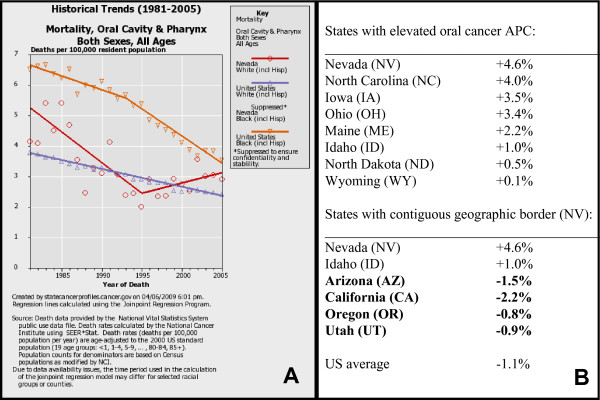
**Analysis of state-specific oral cancer mortality data**. Historical trends (1981-2005) of mortality from oral cancer were sorted by race and ethnicity using NCI SEER*Stat (National Cancer Institute Surveillance Epidemiology and End Results) and regression lines calculated using the Joinpoint Regression Program. A) Oral cancer deaths in Nevada were initially declining, but exhibited a distinct, sustained upward trend among white males beginning in 1995. B) US states previously identified with short-term increases in oral cancer rates were confirmed as NV, NC, IA, OH, ME, ID, ND and WY, while states sharing a contiguous border with Nevada generally experienced declining trends.

Oral cancer rates in those selected states with previously identified increasing APC [9] were recalculated and updated to reveal any changes to the previous trends observed (Figure 1). This re-analysis confirmed the previous report that oral cancer rates have been decreasing in most US states, however a small subset of states have experienced recent increases in rates of death from oral cancer (Figure 1B). This data revision also confirmed the previous report that mortality in the state with the highest APC in oral cancer deaths, Nevada, was decreasing for many years (1981-1995) (Figure 1A). However, the distinct reversal and subsequent upward trend in deaths from oral cancer in Nevada was found to have begun earlier (1995-2005) than previously noted (1998-2004), providing further confirmation this upward trend appears not only to be continuing (2004-2005), but may also be increasing. In addition, this analysis confirmed these observed increases were not among females or minorities, but instead were restricted primarily to white males.

To determine if these trends were restricted to these particular states or if they are part of a larger regional increase, oral cancer rates for US states with contiguous geographic borders to Nevada were analyzed to determine any demonstrable changes (Fig 1B). This analysis revealed the majority of states sharing a contiguous border with Nevada, including Arizona, California, Oregon and Utah, have all experienced decreasing rates of oral cancer deaths, similar to the national US trend. The only state bordering Nevada found to have a positive oral cancer APC was Idaho, a state previously identified as one of the subset of US states with increasing rates of death from oral cancer [9].

### Behavioral Risk Factor Surveillance System (BRFSS)

Epidemiologic evidence has previously demonstrated oral cancer incidence and mortality rates are correlated strongly with two major risk factors, tobacco use - consisting primarily of smoking, and to a lesser extent, heavy alcohol use [10]. To assess the potential relationship between tobacco use, the primary risk factor for oral cancer, and the subset of US states with increasing oral cancer rates, data regarding tobacco use and smoking prevalence in these states was obtained from the Behavioral Risk Factor Surveillance System (BRFSS). Analysis of these data demonstrated that the majority (7/8) of those states with elevated oral cancer APC also had current smoking rates (2007, most current available data) at or above the national average (Table 1). Moreover, all of the states sharing a contiguous border with Nevada, mainly with decreasing rates of oral cancer, were found to have current smoking rates at or below the national average.

**Table 1:** Comparison of smoking rates and tobacco control in selected US states

State	Current rate (2007)	Comparison (relative to US)	IOI (1992-1993)	SoTC (1999-2000)
Elevated APC states:				
NV	21.5%	ABOVE	+0.25	-1.42
NC	22.9	ABOVE	-4.46 (LOW)	-0.14
IA	19.8	SAME	-1.18 (LOW)	**+0.41**
OH	23.1	ABOVE	-2.81 (LOW)	-1.05
ME	20.2	ABOVE	**+1.28 (HIGH)**	-1.24
ID	19.1	**BELOW**	**+1.33 (HIGH)**	**+0.13**
ND	20.9	ABOVE	-0.29	-0.93
WY	22.1	ABOVE	-2.11 (LOW)	-0.92
				
Contiguous border states (NV):				
NV	21.5%	ABOVE	+0.25	-1.42
AZ	19.8	SAME	**+2.99 (HIGH)**	**+4.03**
CA	14.3	**BELOW**	**+4.62 (HIGH)**	**+3.73**
ID	19.1	**BELOW**	**+1.33 (HIGH)**	**+0.13**
OR	16.9	**BELOW**	**+2.70 (HIGH)**	**+0.90**
UT	11.7	**BELOW**	**+4.01 (HIGH)**	-0.29
US average	19.8%	N/A	N/A	mean = 0.0 STD = 1.20

NV (Nevada), NC (North Carolina), IA (Iowa), OH (Ohio), ME (Maine), ID (Idaho), ND (North Dakota), WY (Wyoming), AZ (Arizona), CA (California), OR (Oregon), UT (Utah).

### Initial Outcomes Index (IOI)

Although current smoking rates may indicate state-specific usage for tobacco based on price, availability or other social and economic factors, current oral cancer mortality rates are generally the result of previous smoking prevalence [10]. Recent public health efforts have focused on developing comprehensive, state-specific measures of previous tobacco use through development of a comprehensive index that measured and ranked all US states according to multiple factors, including per capita tobacco consumption, cigarette prices, as well as workplace and home smoking bans. One such comprehensive index or measure of previous smoking prevalence and tobacco control, known as the initial outcomes index (IOI), ranked all US states for these various factors between 1992 and 1993. Analysis of the IOI index data revealed the majority of states with increasing oral cancer APC also earned IOI scores in the LOW or MODERATE categories (6/8), mainly the result of higher rates of smoking and lower rates of tobacco control, such as lower cigarette prices and fewer smoking bans (Table 1). Conversely, all of the states sharing a contiguous border with Nevada, mainly those with decreasing oral cancer rates, earned IOI scores of HIGH - suggesting these states had lower smoking rates and higher overall tobacco controls, including higher cigarette prices and more extensive smoking bans.

### Strength of Tobacco Control (SoTC)

Another comprehensive measure of previous state tobacco usage, known as the strength of tobacco control (SoTC), was subsequently developed by public health officials to rank and compare all US states in 1999 and 2000, similar to the IOI. Once again, an analysis of the SoTC index data revealed the majority of states with increasing oral cancer APC earned negative SoTC scores, with Nevada scoring the lowest (-1.42), suggesting that tobacco control in these states remained comparatively weak and less than the national mean index (mean = 0.0, STD = 1.20) (Table 1). In contrast, all of the states sharing a contiguous border with Nevada had positive SoTC index scores. When combined in this manner, these data provide compelling evidence that the current smoking prevalence in states with elevated oral cancer rates may have long-standing, historical trends of tobacco use and control that may explain, in part, these anomalous state-specific increases in oral cancer.

**Figure 2:**
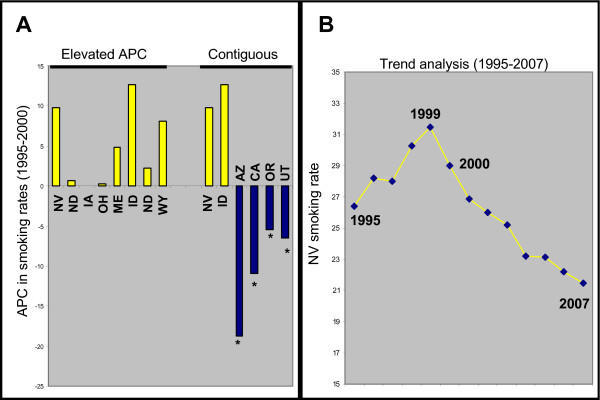
**Analysis of smoking trends in specific US states**. A) Analysis of the annual percent change (APC) or change in smoking trends (1995-2000) from states with elevated oral cancer rates demonstrated these states experienced positive, increasing rates of smoking prevalence, while states sharing a contiguous border with Nevada experienced simultaneous negative or declining rates of smoking, with the exception of Idaho. B) Graphing the smoking prevalence in Nevada revealed a year-by-year increasing trend which peaked in 1999 and subsequently began a steady, sustained decline over successive years.

Although the rates of oral cancer incidence and mortality have declined over the past thirty years, a reversal of these trends has recently emerged during the short-term, which may signify an important change in the epidemiology of this cancer. The IOI, SoTC, as well as current smoking rates provide important information regarding the overall prevalence of tobacco use at specific time points, indicating potential geographic areas that may suffer from tobacco-induced diseases, including oral cancer. A more detailed examination of the changes in short-term trends of tobacco usage rates or smoking prevalence within these states was necessary to explore these potential interactions and effects. Based upon this information, data from BRFSS regarding annual smoking prevalence for states with elevated oral cancer APC and states with a contiguous border to Nevada were assessed to reveal any significant changes (Figure 2).

Detailed analysis of this data revealed that most states with elevated oral cancer APC were found to have increases in the prevalence of smoking during the initial, short-term time period examined (1995 - 2000) (Figure 2A). In addition, most states sharing a contiguous border with Nevada were found to have decreases in the prevalence of smoking over this same time period, with the notable exception of Idaho. To evaluate how these short-term changes in smoking rates may have changed over time, BRFSS smoking prevalence data for all available years (1995-2007) from the individual state with the highest oral cancer APC, Nevada, were obtained and plotted to reveal any significant trends (Figure 2B). This analysis demonstrated that although smoking rates in Nevada were initially increasing between 1995 and 1999, these rates have begun a more recent year-by-year decline - although they remain above the national average.

To determine any changes in smoking prevalence occurring in the states examined so far, BRFSS data for all available years (1995-2007) were obtained and short-term changes in smoking rates were evaluated to uncover any significant trends (Table 2). This analysis revealed that all of the states identified with elevated oral cancer APC also experienced an increase in smoking trends during one or more of the first four time intervals examined (1995-2000, 1996-2001, 1997-2002, 1998-2003). Furthermore, all of the states sharing a contiguous border with Nevada experienced only decreasing rates of smoking during these same intervals, with the exception of Idaho (Table 2). Moreover, these data revealed an important shift and reversal in smoking trends among the states with elevated oral cancer APC during the 1999-2004 interval. This reversal signified a dramatic decrease for each of these states, which has continued during all subsequent intervals (2000-2005, 2001-2006, 2002-2007), albeit by differing percentages.

**Table 2:** Comparison of smoking trends in selected US states

State	1995-2000	1996-2001	1997-2002	1998-2003	1999-2004	2000-2005	2001-2006	2002-2007
Elevated APC states:								
NV	**+9.8**	-4.6	-7.1	-16.8	-26.3	-20.3	-17.4	-17.3
NC	**+0.7**	**0.0**	**+1.9**	**+0.8**	-7.9	-13.4	-14.0	-12.9
IA	0.0	-6.3	**+0.4**	-7.2	-11.4	-12.1	-3.1	-14.6
OH	**+0.3**	-2.8	**+5.9**	-3.4	-6.1	-14.9	-18.8	-13.1
ME	-0.4	-5.5	**+3.9**	**+5.8**	-9.8	-12.6	-12.5	-14.5
ID	**+12.6**	-7.1	**+3.5**	-6.4	-19.1	-19.7	-14.2	-7.2
ND	**+2.2**	-5.5	-3.5	**+2.5**	-9.9	-13.4	-11.7	-2.7
WY	**+8.1**	-9.7	-1.2	**+7.8**	-9.2	-10.5	-2.7	-6.7
								
Contiguous border states (NV):								
NV	**+9.8**	-4.6	-7.1	-16.8	-26.3	-20.3	-17.4	-17.3
AZ	-18.7	-9.2	-10.9	-4.5	-7.9	-8.6	-15.3	-15.3
CA	-10.9	-7.5	-10.8	-12.5	-20.8	-11.6	-13.3	-12.8
ID	**+12.6**	-7.1	**+3.5**	-6.4	-19.1	-19.7	-14.2	-7.2
OR	-5.4	-12.3	-8.2	-0.9	-6.5	-10.6	-9.7	-24.5
UT	-6.5	-4.3	-7.2	-16.1	-25.0	-10.8	-25.7	-8.5

NV (Nevada), NC (North Carolina), IA (Iowa), OH (Ohio), ME (Maine), ID (Idaho), ND (North Dakota), WY (Wyoming), AZ (Arizona), CA (California), OR (Oregon), UT (Utah).

**Figure 3:**
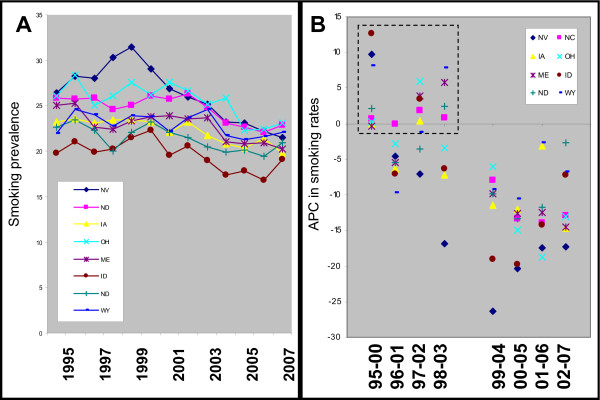
**Analysis of annual smoking prevalence data in states with elevated oral cancer APC**. A) A plot of annual data regarding state smoking prevalence demonstrates some initial variability among varying states, following by a more general declining trend beginning between 1999 and 2001. B) Graphing the trend or five-year annual percent change (APC) from these states revealed the more general trend of variability during the initial time periods (1995-2000 through 1998-2003), that was followed by more general declining trends in subsequent periods (1999-2004 through 2002-2007).

To further examine the changes in smoking prevalence over time from those states with elevated oral cancer APC, BRFSS data for each year were collected and graphed (Figure 3). The year-by-year plot of individual states with elevated oral cancer rates demonstrated that although some initial increases were observed in each state, most states developed a general, decreasing trend that became evident between 1999 and 2001 (Figure 3A). Plotting the smoking prevalence trends from Table 2 to visualize the changes over five-year intervals revealed the dramatic shift from mainly positive trends, or increases in reported state-wide smoking during the first four time intervals to negative trends, or net decreases in smoking during all subsequent time intervals (Figure 3B).

**Figure 4:**
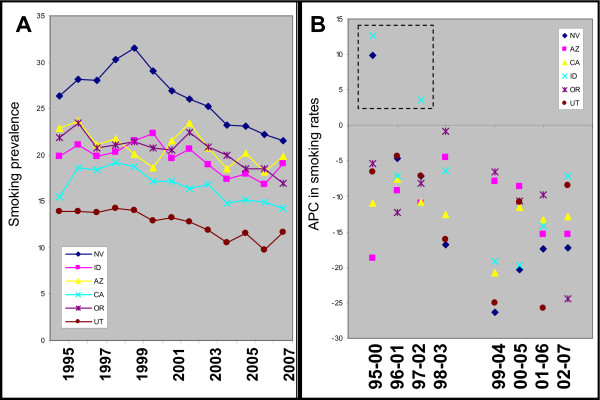
**Analysis of annual smoking prevalence data in states sharing a contiguous border with Nevada**. A) A plot of annual data regarding state smoking prevalence demonstrates a general declining trend for all states during the entire period of available data, with the exception of Nevada, initially. B) Graphing the trend or five-year annual percent change (APC) from these states revealed the general declining trends in all periods (1995-2000 through 2002-2007), with the notable exceptions of Nevada and Idaho.

A similar examination of changes in smoking rates over time was performed using BRFSS data from those states sharing a contiguous border with Nevada to reveal any significant changes in trends and for comparison with those states experiencing elevated oral cancer APC (Figure 4). In detail, the year-by-year plot of smoking trends from these states revealed that most experienced year-by-year decreases for the vast majority of years examined, with the notable exception of Nevada itself (Figure 4A). A plot of the changes in smoking trends for each time period from Table 2 revealed that all of these states experienced declining rates of smoking during all intervals examined (1995-2000, 1996-2001, 1997-2002, 1998-2003, 1999-2004, 2000-2005, 2001-2006, 2002-2007), with the notable exceptions of Nevada and Idaho (Figure 4B).

## Discussion

The overall rates of cancer incidence and mortality have declined within the US in recent decades, but are not uniform or consistent within this population [1-4]. Although strong evidence has shown increased rates among minority groups and women during this same period [5-7], recent evidence has suggested rates are also increasing within particular US states, creating additional health disparities [13]. This study re-examined those data and confirmed that oral cancer rates have increased within this small subset of US states. Moreover, further analysis verified that these trends are not part of larger, regional increases in oral cancer nor are they linked with the previously observed increases among females and minorities, but instead represent state-specific phenomenon with geographic specificity.

Oral cancer has been linked primarily to tobacco use and smoking [10], therefore, this study sought to analyze comprehensive state demographic and behavioral data necessary to reveal the current and historical trends of tobacco use and smoking in these specific states. Although it was expected, and confirmed, that states with higher rates of oral cancer had comparatively higher rates of current, as well as former, smokers than other states, this study exposed more recent, short-term trends that suggest these smoking rates have more recently reversed and are now steadily decreasing over time. Because oral cancer incidence and mortality are generally the result of previous smoking prevalence, this reversal may signify that oral cancer rates within this subset of US states will also begin to decline, although previous observations suggest a lag time of many years [6,7,10,11].

Although epidemiologic studies of demographic and behavioral characteristics provide invaluable methods for identifying subgroups with increased risk for oral cancer within larger populations, this study provides strong evidence of other potential variables, including state-specific indexes of policies and pricing structures for tobacco, that may create "geographic pockets" of increased risk, even among the general population. In addition, the inclusion of workplace and home smoking bans as integral components of the IOI and SoTC indexes may suggest these data have the potential to provide more nuanced and comprehensive measures of state-specific smoking activity and risk than the more commonly reported measures of current adult smokers or per capita cigarette consumption. However, despite recent increases in the number of workplace smoking bans passed in several of these states, the role of second-hand smoke in the work or home environment may represent additional factors that further complicate and exacerbate the effects of tobacco use within these areas [25,26].

Aside from the confounding effects of second-hand smoke, several additional limitations of this study should be noted. For example, some of these states have seen dramatic shifts in population, including a rapid influx of both casino and construction workers in Nevada, which were coupled with an influx of retired and elderly seeking affordable housing in warmer climates [27]. Although the survey and sampling of populations through the CDC, BRFSS and SEER should account for these shifts in population demographics, the possibility remains that these shifts could have skewed the data sampling, which may have resulted in the inaccurate representation of current or former smokers in each state - thereby influencing the outcome of these analyses.

In addition, other potential risk factors for oral cancer have also recently been identified and these underlying medical conditions may have some effects on the different rates observed. For example, immune suppression and immune modulation due to infection with the human immunodeficiency virus (HIV), or by pharmacologic means to prevent rejection of tissue, have increased in prevalence within the US during these same time periods, although their recognized influences on the development of oral cancers have been the subject of relatively fewer epidemiologic investigations [28,29]. Additional evidence that other infectious agents, such as human papillomavirus (HPV), may increase the risk of developing oral cancer and contribute to its progression has also been accumulating [30-33]. Because few data specific to oral HPV prevalence or infection rates are currently available [34], assessing the potential association with increasing rates of oral cancer has remained elusive.

Finally, additional studies examining other modulating factors for oral cancer development have identified potential risk factors that may also influence overall rates, incidence, and mortality. Some studies have demonstrated an inverse relationship between the consumption of fruits or vegetables and oral cancer risk, indicating that dose-dependent reductions in oral cancer risk are possible with each additional serving of fruits or vegetables consumed [35-38]. Moreover, recent epidemiologic evidence has demonstrated that serum and tissue folate levels, highly correlated with fruit and vegetable consumption, may be inhibited by tobacco or alcohol use - known primarily for their direct and indirect carcinogenic effects rather than their modulating effects on micronutrient absorption [39-41]. Although preliminary epidemiologic studies have found inconclusive, and seemingly contradictory, effects of folate status on oral cancer risk [42,43], no studies to date have directly examined the association between folate status and state-specific or demographic increases in oral cancers.

## Conclusion

Due to the rising costs of health care in the US and the limited resources available for health prevention efforts, it is essential to organize and direct more effective efforts by public health officials and epidemiologists, as well as funding from local, state and federal governments, to reduce and eliminate identified health disparities. This study provides evidence of state-specific increases in oral cancer that are not associated with the increases previously observed among females and minorities, thereby providing new insights regarding potential methods to identify changes in relevant trends in geographic areas which may experience increases in tobacco-induced diseases in the future. As state and local public health professionals strive to formulate effective prevention and education programs for their residents, understanding the relationships between cause and effect, as well as the primary or secondary factors that more accurately indicate the potential for increased risk, becomes more imperative.

## Authors' contributions

KK was responsible for the overall design and concept of this study. AB, NP, NR and KS were responsible for data collection and preliminary data analysis. AB and KK were primarily responsible for the writing, while SO and MC were responsible for the majority of editing. All authors have read and approved the final version of this manuscript.

## Declaration of Competing interests

The authors declare that they have no competing interests.
